# A new framework for evaluating the health impacts of treatment for Gaucher disease type 1

**DOI:** 10.1186/s13023-017-0592-6

**Published:** 2017-02-20

**Authors:** Michael L. Ganz, Sean Stern, Alex Ward, Luba Nalysnyk, Martin Selzer, Alaa Hamed, Neal Weinreb

**Affiliations:** 1 Evidera, 500 Totten Pond Road, 5th Floor, Waltham, MA 02451 USA; 2 Evidera, 7101 Wisconsin Avenue, Suite 600, Bethesda, MD 20814 USA; 3 Sanofi Genzyme, 500 Kendall Street, Cambridge, MA 02142 USA; 4 Chatham Decision Sciences, Sarasota, FL 34235 USA; 5 University Research Foundation for Lysosomal Storage Diseases Inc., 7367 Wexford Terrace, Boca Raton, FL 33433 USA

**Keywords:** Gaucher disease, Simulation, Health-related quality-of-life, Economic evaluation

## Abstract

**Background:**

The Disease Severity Scoring System (DS3) is a validated measure for evaluating Gaucher disease type 1 (GD1) severity. We developed a new framework, consisting of health states, transition probabilities between those states, and preferences for those states (utilities) based on the DS3 to predict long-term outcomes of patients starting treatment. We defined nine mutually exclusive (alive) health states based on three DS3 categories: mild (0 ≤ DS3 ≤ 3.5) without symptoms of bone disease; mild with bone pain, mild with severe skeletal complications (SSC) defined as lytic lesions, avascular necrosis, or fracture; moderate (3.5 < DS3 ≤ 6.5) without SSC; moderate with SSC; marked (6.5 < DS3 ≤ 9.5) without SSC; marked with SSC; severe (9.5 < DS3 ≤ 19) without SSC; and severe with SSC. Health-state transition probabilities and utilities were estimated from a longitudinal sample of patients with GD1 who started enzyme replacement therapy (the DS3 Score Study). Age dependent GD1-specific mortality was derived from published data. We used a Markov state-transition model to illustrate how to estimate time spent in each health state.

**Results:**

The average predicted utilities for each health state ranged from 0.76 for mild disease with no clinical symptoms of bone disease to 0.52 with severe disease with SSC. Transition probabilities depended on disease severity (DS3 score) at treatment initiation and whether patients had undergone a total splenectomy or had an intact spleen/partial splenectomy prior to starting treatment. Patients who started treatment with intact or residual spleens spent more time in better health states than those who started treatment with total splenectomy.

**Conclusions:**

This new framework, which is based on the DS3, can be used to project the long-term outcomes of GD1 patients starting treatment. The framework could also be used to compare the long-term outcomes of different GD1 treatment options.

**Trial registration:**

NCT01136304. Registered: May 31, 2010 (retrospectively registered).

**Electronic supplementary material:**

The online version of this article (doi:10.1186/s13023-017-0592-6) contains supplementary material, which is available to authorized users.

## Background

Gaucher disease (GD) is an autosomal recessive lysosomal storage disorder caused by deficient glucocerebrosidase activity [1]. The disease is rare, occurring in about 1 in 100,000 births worldwide and 1 in 850 among Ashkenazi Jews [2]. The most prevalent form is Gaucher disease type 1 (GD1) which accounts for up to 95% of cases [3]. Presentation of the disease is variable, but is typically characterized by visceral problems such as splenomegaly and hepatomegaly, hematologic issues such as anemia and thrombocytopenia, and skeletal complications such as severe bone pain, avascular necrosis, and joint deformities, or loss in bone mineral density with increased risk of bone fragility fractures [1, 4–6].

Current therapeutic options with regulatory approval in either the United States (US) or Europe include intravenous enzyme replacement therapy (ERT) with imiglucerase, velaglucerase alfa, or (in the US only) taliglucerase alfa or oral substrate reduction therapy (SRT) with eliglustat or miglustat [7]. Imiglucerase was licensed in the early 1990s [8] and has been shown to reverse the signs and symptoms of GD1, laboratory and imaging abnormalities, and to reduce the incidence of splenectomy. However, some patients cannot use ERT due to pain, fear of infusions, poor venous access, or side effects, such as hypersensitivity reactions not controllable with pre-medication regimens and neutralizing antibodies. Miglustat, an oral substrate reduction agent approved in Europe (2002) and the US (2003), is recommended for symptomatic patients with mild to moderate clinical manifestations for whom ERT is not an option. Its use is limited, however, due to frequent gastrointestinal side effects, especially diarrhea [9]. Eliglustat, the most recently licensed oral SRT with comparable efficacy to imiglucerase and a favorable safety profile, is approved in the US (2014) and Europe (2015) as a first-line therapy for treatment-naïve and ERT-treated adults with GD1. Although largely free of the gastrointestinal side effects associated with miglustat, eliglustat cannot be prescribed for individuals who are cytochrome P450 2D6 ultra-rapid or indeterminate metabolizers, or patients who are using essential incompatible medications [7]. Eliglustat should be avoided in patients who are pregnant or breast feeding, who have advanced liver, kidney or cardiac disease as it has not been studied in these populations.

Despite significant health-related quality of life (HRQOL) benefits from ERT for patients with GD1 compared with no treatment, and differences in effectiveness and adverse events between ERT and miglustat, little has been published on long-term projected outcomes for these treatments. Studies to date simulating the long-term outcomes of GD1 [8, 10] are inconsistent with current experience by limiting transitions between some states (e.g., patients with certain symptoms could not improve) or else those studies have not explicitly separated information on bone manifestations from hematologic and visceral manifestations nor did they discriminate between less and more severe bone complications, such as bone pain and fractures.

We developed a new framework based on the Disease Severity Scoring System (DS3), a validated measure for evaluating GD1 severity, to address some of the limitations in the current literature [11]. Our framework for describing the health states of patients treated for GD1 focuses on those aspects of treatment that impact patients’ life expectancy, quality-adjusted life expectancy, and time spent in each health state. This article describes this framework, consisting of health states, transition probabilities between those states, and preferences for those states (utilities), and illustrates how it can estimate the long-term outcomes of similar patient cohorts using a Markov state-transition model. This study uses health states based on the DS3 to develop utilities and transition probabilities from a real-world sample of patients diagnosed with GD1 and followed since starting ERT.

## Methods

### Data

The data used for study come from the DS3 Score Study (NCT01136304) that followed 166 eligible patients 18 years and older from four investigative sites in the US and one in Canada; 33 patients were never treated and will be subjects for a future study. Here, we utilized data from the 133 treated patients for whom sufficient data were available to calculate baseline DS3 scores [12]. DS3 scores were calculated for these patients annually from initiation of treatment for an average (standard deviation) of 13.3 (6.1) years. Initially, 57 (42.9%) patients received alglucerase, 73 (54.9%) imiglucerase, 2 (1.5%) velaglucerase, and 1 (0.8%) miglustat. At last follow-up, 105 (78.9%) patients were receiving ERT (71 imiglucerase, 33 velaglucerase, and 1 taliglucerase) and 12 (9.0%) were receiving SRT (6 miglustat and 6 eliglustat); treatment was unknown or had been interrupted in the remainder of patients. ERT was used in 99% of the treated patient-years. All ERT products were assumed to have biosimilar efficacy and therefore interchangeable.

### Health states

We used the DS3 for GD1 to guide the development of health states [11, 12]. The DS3 has been validated for GD1 patients at least 18 years old and was designed to capture the heterogeneous and dynamic aspects of the disease, especially skeletal complications that clinicians consider important when assessing disease severity. The DS3 was developed to serve as a standardized instrument to measure GD1 symptoms and severity (burden of disease) and to classify cohorts in GD1 clinical studies. The DS3 has three domains that include bone disease/skeletal, hematologic, and visceral symptoms. Each domain is measured by three or more assessment items scored by a physician. Content, face, criterion, discriminant, construct, and feasibility validity were deemed high by global GD1 experts and physicians treating patients with GD1 [11]. The DS3 is highly correlated with other severity measures, such as the Clinical Global Impression (CGI) scale, the CGI-Severity (CGI-S), and the Zimran Severity Score Index [13, 14]. Summary information on scoring the DS3 is available in Additional file 1: Appendix 1.

The nine mutually exclusive (alive) health states are based on GD1 severity categories defined by the total DS3 score category (mild, moderate, marked, and severe) and the presence or absence of bone pain (BP) or severe skeletal complications (SSC), which include lytic lesions, avascular necrosis, or fracture: (1) mild (0 ≤ DS3 ≤ 3.5) with no clinical symptoms of bone disease, (2) mild with BP, (3) mild with SSC, (4) moderate (3.5 < DS3 ≤ 6.5) without SSC, (5) moderate with SSC, (6) marked (6.5 < DS3 ≤ 9.5) without SSC, (7) marked with SSC, (8) severe (9.5 < DS3 ≤ 19) without SSC, and (9) severe with SSC. (This rank ordering was used in the ordinal regression analysis described below to derive transition probabilities.) Thus, each health state has two dimensions to best capture a treatment’s impact on hematologic and visceral symptoms and on bone disease and to create homogeneous groups of patients within each health state. Even though we do not conceptualize it as a health state, splenectomy status can still influence health outcomes in two ways: via its impact on the DS3 score, which defines the health state, and via its impact on health-state transition probabilities (as described below).

We estimated health state-specific utilities from patients enrolled in the DS3 Score Study for whom responses from the SF-36 (Version 1) questionnaire were available [15]. DS3 and SF-36 data were not collected consistently or at fixed time intervals in the DS3 Score Study. As a result, only patient DS3 scores, and their corresponding health states, that could be matched to (the closest) SF-36 responses within a 90-day window around the DS3 score measurements were used to derive utilities. The original SF-36 measures were converted to the EuroQoL EQ-5D [16] utilities for the United Kingdom (UK) population using the method developed by Brazier and Roberts (2004) [17].

We assumed that health-state utilities depended on the DS3 severity category, bone pain, the presence of SSC, and patients’ age and sex at treatment initiation as follows:

$$ U= f\left( D, B, S, A, X\right), $$

where *U* represents utility values, *D* the DS3 severity (mild, moderate, marked, severe), *B* the presence of bone pain, *S* the presence of SSC, *A* the age when the patient started treatment, and *X* the patient’s sex. This equation was fitted with the generalized estimating equation method with a Gaussian error term and the identity link function, to account for multiple observations per patient, using data from the DS3 Score Study; heteroscedasticity-consistent (robust) standard errors were estimated. Utilities were calculated from the estimated regression coefficients and the values of *D*, *B*, and *S* corresponding to each health state using the method of recycled predictions [18]. The effect of bone pain was included when calculating utilities for health states that included SSC (see Additional file 2: Appendix 2).

### Transitions between health states

Patients can remain in the same health state, transition to more or less severe health states, or die. We assumed that a patient’s probability of being in a particular health state, except for death, at a particular time depends on the health state in the previous period, the length of time the patient was receiving treatment, and other clinical characteristics as follows:

$$ {\mathrm{P}}_{\mathrm{r}}\left({H}_t\right)= f\left({H}_{t-1},{T}_t,,, D, S\right) $$

where *H* is one the nine health states described above, *T* is treatment duration (1, 2, or ≥ 3 years), *D* is the starting DS3 category (mild, moderate, marked, or severe), *S* is the patient’s splenectomy status, and *t* indexes the time period. We included *T* in the equation to capture the effect of disease stabilization over time [19, 20].

This equation was fitted with the ordered logistic regression method using data from the DS3 Score Study; heteroscedasticity-consistent (robust) standard errors were estimated. Health states for the dependent variable were rank ordered based on the nine disease states described above. Although the assumption of proportional odds between categories of the dependent variable was not satisfied, it was not possible to use the less restrictive multinomial logistic or other more robust methods due to sparse data. Twenty-four annual health-state transition probability matrices were calculated from the estimated ordered logistic regression coefficients and all combinations of *T* (3 levels) *D* (4 levels), and *S* (2 levels) using the method of recycled predictions [18] (see Additional file 2: Appendix 2).

### Model overview and analysis

We implemented our framework as a Markov state-transition model in Microsoft Excel® to simulate the experiences of cohorts of patients with GD1. The model includes nine health states based on DS3 Score Study severity categories as well as an absorbing state for death. Patients are assigned state-specific utilities for each health state. Except for death, patients can transition between health states, or remain in the same state, between each annual cycle. Although patients may have undergone splenectomy prior to starting the model, we assumed, in accordance with current clinical guidelines, that patients will not undergo a splenectomy once they start receiving treatment [21]. Total undiscounted and discounted (3.5% per annum) quality-adjusted life years (QALYs) are calculated and a half-cycle correction is applied.

This model also includes death as an absorbing state. We estimated age-specific GD1 mortality probabilities from summary mortality data on patients enrolled in the International Collaborative Gaucher Group (ICGG) Gaucher Registry using a Gompertz survival function; the overall GD1 life expectancy at birth was 68 years (64 years for splenectomized patients and 72 years for nonsplenectomized patients) [22]. Because the estimated mortality risks for ages 77 years and older were smaller than the corresponding mortality risks for the general UK population, we applied the maximum of the GD1-specific and the UK general population (http://www.mortality.org) mortality risks for any given age to the patients in the model (see Additional file 2: Appendix 2).

To show how transition probabilities and utilities are used to predict long-term outcomes we focused on patients in the three most common health states observed in the DS3 Score Study data: mild with no clinical symptoms of bone disease (19%), moderate without SSC (28%), and marked with SSC (28%). The analyses were stratified by splenectomy status. Consistent with the characteristics of the patients enrolled in a recent clinical trial (ENGAGE), we assumed that 50% of the simulated cohort were women and all started treatment at 32 years of age [23]. Total undiscounted and discounted (3.5% per annum) quality-adjusted life years (QALYs) are calculated and a half-cycle correction is applied.

## Results

### Health state utilities

Table 1 displays the estimated coefficients of the health state regression equation. We found that the three broad severity categories of moderate, marked, and severe had increasingly larger negative effects on health-state utility compared with the mild severity category. Bone pain was also negatively related to utility.

**Table 1:** Generalized Estimating Equation Regression Results for Health State Utility in Gaucher Disease Type 1

	Mean or Proportion for estimation sample	Coefficient	Robust standard error	95% CI
DS3 Severity (vs. Mild)				
Moderate	0.381	−0.078**	0.037	−0.150, −0.005
Marked	0.113	−0.122***	0.042	−0.205, −0.039
Severe	0.021	−0.168**	0.055	−0.275, −0.061
Bone Pain	0.258	−0.098***	0.034	−0.165, −0.032
Severe Skeletal Complications	0.103	0.018	0.037	−0.055, 0.090
Female	0.691	−0.049	0.030	−0.108, 0.010
Age at Treatment Initiation	53.9	−0.002*	0.001	−0.004, 0.001
Constant		0.880***	0.078	0.727, 1.033
Number of Observations	97			
Number of Patients	50			

***p < 0.01, **p < 0.05, *p < 0.10

*Abbreviations: CI* confidence interval, *DS3* disease severity scoring system

**Fig. 1:**
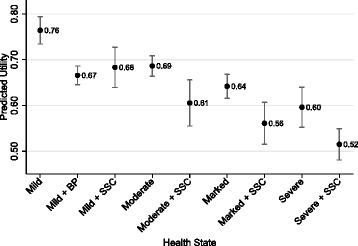
Predicted Health State Utilities in Gaucher Disease Type 1. *Note*: Error bars correspond to robust 95% confidence intervals

Figure 1 displays the average predicted utilities for each health state, which reflects the substantial impact of GD1 on HRQOL, especially due to skeletal complications [24, 25]. The utility values for patients with GD1 depend on disease severity and ranged from 0.76 for patients with mild disease and no clinical symptoms of bone disease to 0.52 for patients with severe disease with SSC; six of the nine utility values were between 0.60 and 0.69. These estimated utilities are all substantially below the EQ-5D population norm for the United Kingdom of 0.86 [26].

### Transition probabilities

The estimated coefficients of the health state transition regression equation are presented in Table 2. Patients in the moderate, marked with and without SSC, and severe with and without SSC states were much more likely to be in health states worse than mild in the next time period than patients in the mild with BP, mild with SSC, and moderate with SSC states. Patients with intact spleens were less likely to transition to health states worse than mild than those who had undergone a splenectomy.

**Table 2:** Ordered Logistic Regression Results for Annual Health-State Transitions in Gaucher Disease Type 1

	Proportion for estimation sample	Coefficient	Robust standard error	95% CI
Health State in Previous Year (vs. Mild)				
Mild + Bone Pain	0.114	1.305***	0.241	0.833, 1.777
Mild + SSC	0.010	0.840*	0.452	−0.046, 1.727
Moderate	0.344	2.581***	0.305	1.983, 3.179
Moderate + SSC	0.041	1.253***	0.381	0.506, 2.000
Marked	0.028	4.504***	0.465	3.593, 5.416
Marked + SSC	0.062	3.617***	0.462	2.711, 4.524
Severe	0.003	4.213***	0.698	2.845, 5.581
Severe + SSC	0.007	6.072***	1.374	3.380, 8.765
Years on Treatment (vs. One Year)				
Two Years	0.086	0.293	0.361	−0.414, 000
Three or More Years	0.807	0.315	0.305	−0.283, 0.913
Baseline DS3 Category (vs. Mild)				
Moderate	0.312	−0.150	0.276	−0.691, 0.391
Marked	0.410	0.873***	0.274	0.336, 1.410
Severe	0.071	1.349***	0.411	0.543, 2.154
Not Splenectomized	0.672	−1.089***	0.250	−1.578, −0.599
Ordered Logistic Cutpoints				
Cutpoint 1		0.740	0.456	−0.153, 1.634
Cutpoint 2		1.662***	0.464	0.752, 2.572
Cutpoint 3		1.727***	0.467	0.812, 2.641
Cutpoint 4		5.054***	0.519	4.037, 6.070
Cutpoint 5		5.835***	0.523	4.810, 6.861
Cutpoint 6		6.577***	0.633	5.335, 7.818
Cutpoint 7		8.808***	0.639	7.555, 10.060
Cutpoint 8		9.066***	0.650	7.792, 10.340
Number of Patients	130			
Number of Observations	970			

Standard errors account for multiple observations per patient. *** *p* < 0.01, **p < 0.05, *p < 0.10

*Abbreviations*: *CI* confidence interval, *SSC* severe skeletal complications

We display, as examples, the transition probability matrices for patients who have and have not undergone a splenectomy prior to starting treatment (*S* = 0, 1) in Tables A1 and A2, respectively, in Additional file 2: Appendix 2. These four probability matrices, which were computed assuming *T* = 1 and *D* = mild and *T* = 1 and *D* = moderate, indicate that a considerable proportion of patients transition to less severe states, based on the rank ordering described earlier (the probabilities below and to the left of the diagonals in each table are typically greater than the probabilities above and to the right) and that the probabilities of improving are greater for patients with intact spleens compared with those who have been splenectomized. The 20 transition probability matrices based on the other values of *T*, *D*, and *S* are available upon request.

### Projected long-term outcomes

Table 3 displays the quality-adjusted life years accrued by the hypothetical cohorts in our model and the corresponding proportions of total time spent in each health state. Patients who started in the mild or moderate state accrued almost one more discounted QALYs than patients who started in the marked with SSC state.

**Table 3:** Model Results (Projected Lifetime Outcomes) in Gaucher Disease Type 1

	Starting Health State					
	Intact Spleen			No Spleen		
	Mild	Moderate	Marked with SSC	Mild	Moderate	Marked with SSC
Discounted QALYs	15.73	15.69	14.92	15.70	15.65	14.89
Undiscounted QALYs	31.16	31.20	29.73	31.13	31.16	29.71
% of Remaining Lifetime Spent in:						
Mild	69.72	70.61	33.86	68.94	69.57	19.55
Mild with BP	12.86	12.26	16.13	13.15	12.23	13.37
Mild with SSC	0.73	0.69	1.17	0.75	0.70	1.07
Moderate	15.69	15.58	42.20	16.14	16.50	52.97
Moderate with SSC	0.53	0.46	2.76	0.55	0.54	5.40
Marked	0.24	0.21	1.36	0.25	0.24	3.01
Marked with SSC	0.20	0.17	2.38	0.21	0.20	3.02
Severe	0.01	0.00	0.03	0.01	0.01	1.28
Severe with SSC	0.02	0.02	0.12	0.02	0.02	0.33

Results for cohorts starting treatment at age 32 (patients in all cohorts have 42.29 remaining life years). QALYs discounted 3.5% per annum

Patients with intact spleens who started treatment in the mild or moderate state spent more of their lifetimes in time in the mild to moderate states (about 99%) than patients who started treatment in the marked plus SSC state (about 94%). Similar patterns are seen for patients who were splenectomized, except that those who started treatment in the marked plus SSC state spent less of their lifetimes in the mild state (about 20%) than those who started treatment in the marked plus SSC state with intact spleens (about 34%).

We compared the distribution of health states over time predicted by the model to the actual experiences of the patients in the DS3 Score Study. Table A3 in Additional file 2: Appendix 2 displays these distributions for patients starting treatment in the mild, moderate, and marked with SSC health states. Due to sample size limitations, we only compared patients who had not undergone splenectomy. Overall, the predictions matched the observed data reasonably well given the limited sample size, but the predictions did deviate from the observed data in some cases.

## Discussion

This research presents a new framework to describe the course of GD1 for treated patients. This framework extends the literature by presenting newly defined GD1 health states, their corresponding utility values, and health-state transition probabilities based on the DS3 that were derived from a single real-word sample followed since starting ERT. The Markov model uses these health states and parameters to simulate the long-term outcomes of three cohorts upon starting treatment.

Our newly defined health states are based on the DS3, a measure developed to specifically assign weights to the aspects of GD1 that clinicians consider important when assessing disease severity. These health states extend previous work [8, 10] by showing that patients’ health status and HRQOL can deteriorate *or* improve over time and that these transitions are influenced by splenectomy status and disease severity when starting treatment. These health states also capture the hematologic and visceral dimensions of the disease separately from the bone dimension, which help to characterize the disease and its response to treatment. For example, Charrow and Scott emphasized that hematologic parameters, liver and spleen volumes, and bone disease do not necessarily respond to treatment to the same extent or at the same time and that response may be affected by timing of treatment initiation, enzyme dose, and the presence of irreversible complications such as osteonecrosis [27]. Deegan et al. and Weinreb et al. have also shown that patients can still experience significant bone complications after starting ERT [12, 28], even as overall disease burden, measured by the DS3, has decreased over time [12].

We found that treated GD1 patients reported lower levels of HRQOL as measured by EQ-5D utilities, than the general population and that those utilities ranged from 0.76 for the mild health state to 0.52 for severe with SSC. Although these estimated utility values are consistent with the study by Deegan et al. that reported a lower median EQ-5D values (0.68 and 0.63) for patients who ever had osteonecrosis or who suffered a frailty fracture than those who had neither (0.80) despite ongoing ERT [28], our values tend to be lower than those reported by van Dussen et al., which ranged from 0.72 to 0.93, except for malignancy, which was 0.15 [8]. Our values may be lower than those reported by van Dussen et al. for a number of reasons: our states may already include attributes of the states used by van Dussen et al., the characteristics of the samples were different, the direct use of the EQ-5D by van Dussen et al. rather than our use of EQ-5D utilities mapped from responses to the SF-36, and different recall periods inherent in the HRQOL instruments used. It should be noted, however, that the difference between the asymptomatic and bone complications health states (−0.07) reported by van Dussen et al. was similar to the difference we observed due to SSC (−0.08).

Utilities for health states involving bone complications were typically lower than utilities for health states without bone complications. For example, the average utilities for the mild and moderate health states were 0.76 and 0.67, whereas they were 0.67 and 0.61 for the mild with BP and moderate with SSC states. These utilities can be used in future economic evaluations to calculate QALYs of competing GD1 therapies. We also have estimated a function that predicts transition probabilities among these health states conditional on time on treatment, disease severity when starting treatment, and splenectomy status that is consistent with other studies of the course of the GD1, onset of action, and duration of treatment effect [19, 20, 29–32]. This function can be used to derive the transition probability matrices needed to project short- and long-term outcomes of patients after starting treatment.

The results presented here should be interpreted in light of the limitations of this research. Although this framework is meant to capture the most salient clinical aspects of GD1, it does simplify a complex disease and does not capture disease progression for patients not receiving treatment. The health states may have been different if we based them on another validated GD1 severity measure. However, other measures, such as the Gaucher Disease Severity Index-Type I [33], may not be as readily available or easy to use in clinical practice and research studies as the DS3. The uncertainty surrounding the point estimates for the health-state utilities are due, in part, to the relatively small numbers of patients observed in some health states and the way in which they were estimated. Rather than eliciting EQ-5D utility values directly from patients, the utilities were derived from SF-36 scores, which were not always measured at the same time that DS3 scores were assessed. Similarly, methods other than the ordered logistic regression approach may have produced different health-state transition probabilities. The data presented here are based on patients who were predominantly treated with ERT (imiglucerase in about half the cases). The extent to which the utility values results differ for other treatments is unclear in the absence of information on the disutility associated with intravenous therapy. Furthermore, without data from head-to-head studies assessing the efficacy of imiglucerase compared with other treatments, as well as of different doses and administration intervals, we do not know if the transition probabilities differ for other treatments. Finally, because this framework was developed to compare different treatment options, it lacks data on the (untreated) natural history of GD1.

Despite these limitations, the framework and data presented here contribute to the literature evaluating GD1 treatments by providing new information on the predicted long-term clinical outcomes of patients after starting treatment. This framework can be updated to accommodate data on the relative real-world efficacy of treatment options, such as eliglustat [23, 34, 35], as they become available. Although we did not include the use of healthcare services, and their associated costs, or other aspects of treatment, such as adverse events and treatment discontinuation/switching, these factors can be included in a full cost-effectiveness analyses within a local context.

## Conclusions

We have developed a new framework that can be used to project the long-term outcomes of GD1 patients starting treatment. This framework, along with the data presented here, is comprised of newly defined GD1 health states, and their corresponding utility values, and health-state transition probabilities based on the DS3. This framework can also be used to evaluate multiple GD1 treatments and can be updated to accommodate data on the relative real-world efficacy of GD1 treatment options.

## Additional files

Additional file 1: Appendix 1. Gaucher Disease Type 1 Severity Scoring System (GD-DS3). (DOCX 67 kb)
Additional file 2: Appendix 2. Estimating health state utilities, transition probabilities, and mortality risk. (DOCX 40 kb)

## Abbreviations

BP: Bone pain
DS3: Disease severity scoring system
ERT: Enzyme replacement therapy
GD: Gaucher disease
GD1: Gaucher disease type 1
HRQOL: Health-related quality of life
ICGG: International Collaborative Gaucher Group
QALY: Quality-adjusted life year
SRT: Substrate reduction therapy
SSC: Severe skeletal complications
UK: United Kingdom
