# Supplementary material for: A new framework for evaluating the health impacts of treatment for Gaucher disease type 1
Source: Orphanet J Rare Dis. 2017 Feb 20;12:38. doi: 10.1186/s13023-017-0592-6 (PMC5319149; doi:10.1186/s13023-017-0592-6)
Supplement: Additional file 1: Appendix 1. — Gaucher Disease Type 1 Severity Scoring System (GD-DS3). (DOCX 67 kb) [file 13023_2017_592_MOESM1_ESM.docx]

**A New Framework for Evaluating the Health Impacts of Treatment for Gaucher Disease Type 1**

M. L. Ganz, S. Stern, A. Ward, L. Nalysnyk, M. Selzer, A. Hamed, N. Weinreb

# Appendix 1

**Gaucher Disease Type 1 Severity Scoring System (GD-DS3)**


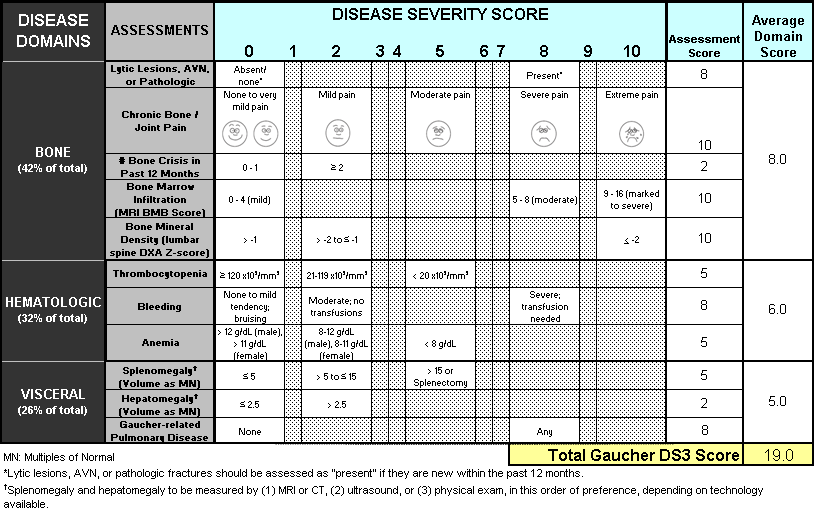


Refer to the following for further details.

1. Weinreb NJ, Cappellini MD, Cox TM, Giannini EH, Grabowski GA, Hwu WL, Mankin H, Martins AM, Sawyer C, vom Dahl S, et al. A validated disease severity scoring system for adults with type 1 Gaucher disease. Genet Med. 2010;12(1):44-51. doi: 10.1097/GIM.0b013e3181c39194

2. Weinreb NJ, Finegold DN, Feingold E, Zeng Z, Rosenbloom BE, Shankar SP, Amato D. Evaluation of disease burden and response to treatment in adults with type 1 Gaucher disease using a validated disease severity scoring system (DS3). Orphanet J Rare Dis. 2015;10:64. doi: 10.1186/s13023-015-0280-3
