# Supplementary material for: A new framework for evaluating the health impacts of treatment for Gaucher disease type 1
Source: Orphanet J Rare Dis. 2017 Feb 20;12:38. doi: 10.1186/s13023-017-0592-6 (PMC5319149; doi:10.1186/s13023-017-0592-6)
Supplement: Additional file 2: Appendix 2. — Estimating health state utilities, transition probabilities, and mortality risk. (DOCX 40 kb) [file 13023_2017_592_MOESM2_ESM.docx]

**A New Framework for Evaluating the Health Impacts of Treatment for Gaucher Disease Type 1**

M. L. Ganz, S. Stern, A. Ward, L. Nalysnyk, M. Selzer, A. Hamed, N. Weinreb

# Appendix 2

## Estimating Health State Utilities from Generalized Estimating Equation Coefficients

We used the “xtgee” command in Stata 11.2 to estimate the utility regression model (standard errors were adjusted for multiple observations per patient using the “robust” option in Stata):

xtgee U i.D B S A F, family(gaussian) link(id) robust

where F is one for female and zero for male. The i. symbol indicates that the variable should be treated as a factor, rather than a continuous, variable.

The estimated regression coefficients can be used to predict the utility for the values of $D$, $B$, and $S$ that define each health state using the following formula:

$U_{h}=Z_{h}\beta_{z}+\bar{X}\beta_{x}$,

where $Z_{h}$ is the vector containing unity and the values $D$, $B$, and $S$ that define health state $h$; $\beta_{z}$ is the vector containing the intercept and the coefficients for $D$, $B$, and $S$; $\bar{X}$ is the vector containing the average values of $A$ and $F$; $\beta_{x}$ is the vector containing the coefficients for $A$ and $F$; and $N$ is the number of observations. The effect of bone pain was included in the utilities for health states that included SSC. In other words, we set $B=\text{yes}$ for health states that included SSC (where $S=\text{yes}$). Utilities were computed for each combination of $D$ (mild, moderate, marked or severe), $B$ (no or yes), and $S$ (no or yes) using Stata’s margins command:

* Utilities for mild

margins, at(D=(1) B=(`0') S=(`0')

margins, at(D=(1) B=(`1') S=(`0')

margins, at(D=(1) B=(`1') S=(`1')

* Utilities for moderate-severe

forvalues t=2/4{

margins, at(D=(`d’) B=(`0') S=(`0')

margins, at(D=(`d’) B=(`1') S=(`1')

}

## Deriving Transition Probabilities from Ordered Logistic Regression Coefficients

We pooled all of the observations from all of the patients in the DS3 Score Study data except those (1) whose clinical assessments were made before starting treatment, (2) who were missing a DS3 score (and hence the health state), or (3) who were missing information on when they initiated treatment. We divided each patient’s follow-up time in the DS3 Score Study database into years (12-month intervals) starting with each patient’s date of treatment initiation. The first 12 months that a patient was using treatment was defined as Year 0, the second 12 months (months 13–24) were defined as Year 1, and so forth.

Patients were observed at multiple time points that may not have exactly corresponded to the 12-month intervals defined above. As a result, 34% of patients had multiple observations in the same year. Because multiple observations from the same patient in the same health state in the same year do not contribute additional information about health-state transitions, we removed all but one observation for patients with multiple observations in the same health state within the same 12-month period. However, if patients were observed multiple times in a given year in different health states, we retained only the observation associated with the worst health state. We used this conservative approach as a way to capture transitions between health states that otherwise may have gone undetected in our analyses because most of the time patients were in less severe health states. Because we needed to estimate annual transitions, i.e., transitions from Year $t$ to Year $t+1$ we included only observations from consecutive years. For example, observations for Years 1, 2, 3, and 4 for a given patient would be retained for the analyses, but only Years 3 and 4 would be retained if data for Year 1 (or Year 2) were unavailable for that patient.

We used the “ologit” command in Stata 11.2 to estimate the regression model (standard errors were adjusted for multiple observations per patient using the “cluster” option in Stata):

ologit H i.H_lag i.T i.D i.S, cluster(p)

where H measures the health status at time $t$, H_lag measures the health status at time $t-1$ for the same patient, and p is an anonymous patient identifier variable. The i. symbol indicates that the variable should be treated as a factor, rather than a continuous, variable.

The estimated ordered logistic regression coefficients can be used to predict the probabilities of being in a particular health state using the following formulas, where $X$ is the vector containing the lagged health status, time on treatment, baseline severity, and splenectomy status variables:

$\Pr\left( H=1 \right)=\left( 1+exp(X\beta-k_{1}) \right)^{-1}$,

$\Pr\left( H=h \right)=\left( 1+exp(X\beta-k_{h}) \right)^{-1}-\left( 1+exp(X\beta-k_{h-1}) \right)^{-1}$ for $H = 2, ..., 8$, and

$\Pr\left( H=9 \right)=1-\left( 1+exp(X\beta-k_{8}) \right)^{-1}$,

where $H$ represents the health state, $\beta$ represents the regression coefficients, $X$ represents the collection of covariates, $k_{h}$ represents the threshold values on the probability distribution for $H$ that separate one discrete health state from another (these coefficients are also referred to as “cutpoints”), and the numbers $1, \ldots, 9$ index the health states mild through severe with SSC in the same order as presented in the “Health States” section of the article.

Transition probability matrices were computed for each combination of $T$ (on treatment for 1, 2, or ≥ 3 years), $D$ (mild, moderate, marked, or severe baseline DS3 category), and $S$ (nonsplenectomized or splenectomized) using Stata’s margins command:

forvalues t=1/3{

forvalues s=0/1{

forvalues d=1/4{

forvalues h=1/9{

margins, at(H_lag=(1/9) T=(`t') S=(`s') D=(`d')) predict(outcome(`h'))

}

}

}

}

## Mortality

We derived the GD1-specific mortality function using information on number of deaths and person-years of follow-up in Table 2 of Weinreb et al. (Am. J. Hematol. 2008;83:896–900) in two steps. First, we derived survival probabilities for each age group and used those probabilities to generate simulated patient-level (SPL) data. Second, we estimated the Gompertz survival function from the SPL data. These steps are described below.

### Deriving Survival Probabilities and Generating SPL

We derived the incidence rate (IR), which approximates the hazard rate (HR), from the number of deaths and follow-up times in Table 2 of Weinreb et al. (2008), for each age group $t\geq1$. We then estimated the survival probabilities, $S(t)$ for each age group as follows:

$$S\left( t \right)=\text{exp}\left( \ln\left( S\left( t-1 \right) \right)-\text{HR}_{t} \right).$$

We assumed 0% mortality (100% survival) for the first age group (0 to < 1 years). Therefore, $S(0)=1$ and subsequent survival probabilities were iteratively computed using the incidence rates as estimates of the hazard rates. We generated SPL data (simulated ages of death and censoring) according to the method described by Tierney et al. (Trials. 2007;8:16). The Kaplan-Meier curves derived from the survival probability function and the SPL data were almost identical.

### Estimating Survival and Mortality Risk

Finally, we fit a number of parametric survival functions (exponential, Weibull, Gompertz, log-logistic, and log-normal) to the SPL data. We assessed the fit of each function by examining the clinical plausibility of the distributions of the projected survival curves and median estimates, the Akaike information criterion and Bayesian information criterion fit statistics, and diagnostic plots. These diagnostics suggested that the Gompertz parameterization is the most adequate fit for these data. The Gompertz parameters are displayed below.

|  | Coefficient | Standard Error |
| --- | --- | --- |
| Intercept | 6.7954 | 0.0814 |
| Gamma | 0.0446 | 0.0014 |

The Gompertz survival probabilities at each age $t$ were computed as $S\left( t \right)=exp\left\{ \left( 1-\exp\left( \gamma t \right) \right)\lambda/\gamma\right\}$ where $\lambda=\text{exp(}\text{}\text{Intercept)}$. The GD1-specific mortality risks ($M^{G}$) at each age $t$ were derived from the Gompertz survival probabilities using the formula $M_{t}^{G}=\left( S\left( t-1 \right)-S\left( t \right) \right)/S(t-1)$.

The estimated mortality risks for ages 77 years and older were smaller than the corresponding mortality risks for the general population. As noted in the Figure in Weinreb et al. (2008), GD1 life expectancy is lower at every age for GD1 patients than it is for the general population. Therefore, we applied the maximum of the GD1-specific and the UK general population mortality probabilities at each age $t$ in the model. In other words, the mortality risk at each age $t$ was computed as $\text{max}\left( M_{t}^{G},M_{t}^{U} \right)$, where$M_{t}^{U}$, the age-specific the general UK population mortality risks, were derived from life tables. It should be noted that the GD1 mortality data we used are over 12 years old at this point. Life expectancy has increased for nearly all Western general populations and, thus, the GD1 mortality risks presented here may be somewhat overestimated.

## Table A1. Health-State Transition Probabilities in Gaucher Disease Type 1, Mild

$T=1$, $D=\text{Mild}$, $S=\text{Nonsplenectomized}$

| **From↓ To🡪** | **Mild** | **Mild with BP** | **Mild with SSC** | **Moderate** | **Moderate with SSC** | **Marked** | **Marked with SSC** | **Severe** | **Severe with SSC** |
| --- | --- | --- | --- | --- | --- | --- | --- | --- | --- |
| **Mild** | 0.8617 | 0.0783 | 0.0035 | 0.0544 | 0.0012 | 0.0005 | 0.0004 | 0.0000 | 0.0000 |
| **Mild with bone/joint pain** | 0.6282 | 0.1812 | 0.0098 | 0.1730 | 0.0042 | 0.0019 | 0.0015 | 0.0000 | 0.0001 |
| **Mild with SSC** | 0.7289 | 0.1422 | 0.0071 | 0.1169 | 0.0027 | 0.0012 | 0.0010 | 0.0000 | 0.0001 |
| **Moderate** | 0.3204 | 0.2219 | 0.0160 | 0.4141 | 0.0148 | 0.0067 | 0.0055 | 0.0002 | 0.0005 |
| **Moderate with SSC** | 0.6401 | 0.1771 | 0.0095 | 0.1659 | 0.0040 | 0.0018 | 0.0015 | 0.0000 | 0.0001 |
| **Marked** | 0.0645 | 0.0831 | 0.0083 | 0.6814 | 0.0810 | 0.0410 | 0.0361 | 0.0010 | 0.0035 |
| **Marked with SSC** | 0.1433 | 0.1527 | 0.0136 | 0.6163 | 0.0388 | 0.0182 | 0.0153 | 0.0004 | 0.0014 |
| **Severe** | 0.0844 | 0.1037 | 0.0101 | 0.6750 | 0.0645 | 0.0316 | 0.0273 | 0.0008 | 0.0026 |
| **Severe with SSC** | 0.0142 | 0.0207 | 0.0022 | 0.4805 | 0.1834 | 0.1301 | 0.1476 | 0.0048 | 0.0166 |

$T=1$, $D=\text{Mild}$, $S=\text{Splenectomized}$

| **From↓ To🡪** | **Mild** | **Mild with BP** | **Mild with SSC** | **Moderate** | **Moderate with SSC** | **Marked** | **Marked with SSC** | **Severe** | **Severe with SSC** |
| --- | --- | --- | --- | --- | --- | --- | --- | --- | --- |
| **Mild** | 0.6771 | 0.1634 | 0.0085 | 0.1447 | 0.0034 | 0.0015 | 0.0012 | 0.0000 | 0.0001 |
| **Mild with bone/joint pain** | 0.3625 | 0.2258 | 0.0156 | 0.3731 | 0.0123 | 0.0056 | 0.0046 | 0.0001 | 0.0004 |
| **Mild with SSC** | 0.4751 | 0.2195 | 0.0135 | 0.2773 | 0.0079 | 0.0035 | 0.0029 | 0.0001 | 0.0003 |
| **Moderate** | 0.1370 | 0.1481 | 0.0134 | 0.6237 | 0.0406 | 0.0191 | 0.0161 | 0.0004 | 0.0015 |
| **Moderate with SSC** | 0.3745 | 0.2262 | 0.0154 | 0.3620 | 0.0117 | 0.0053 | 0.0043 | 0.0001 | 0.0004 |
| **Marked** | 0.0227 | 0.0324 | 0.0035 | 0.5755 | 0.1570 | 0.0972 | 0.0985 | 0.0030 | 0.0103 |
| **Marked with SSC** | 0.0533 | 0.0707 | 0.0072 | 0.6768 | 0.0940 | 0.0488 | 0.0438 | 0.0013 | 0.0043 |
| **Severe** | 0.0301 | 0.0422 | 0.0045 | 0.6218 | 0.1365 | 0.0789 | 0.0760 | 0.0023 | 0.0077 |
| **Severe with SSC** | 0.0048 | 0.0072 | 0.0008 | 0.2525 | 0.1757 | 0.1824 | 0.3156 | 0.0132 | 0.0477 |

## Table A2. Health-State Transition Probabilities in Gaucher Disease Type 1, Moderate

$T=1$, $D=\text{Moderate}$, $S=\text{Nonsplenectomized}$

| **From↓ To🡪** | **Mild** | **Mild with BP** | **Mild with SSC** | **Moderate** | **Moderate with SSC** | **Marked** | **Marked with SSC** | **Severe** | **Severe with SSC** |
| --- | --- | --- | --- | --- | --- | --- | --- | --- | --- |
| **Mild** | 0.8786 | 0.0693 | 0.0031 | 0.0472 | 0.0010 | 0.0004 | 0.0004 | 0.0000 | 0.0000 |
| **Mild with bone/joint pain** | 0.6625 | 0.1690 | 0.0089 | 0.1529 | 0.0037 | 0.0016 | 0.0013 | 0.0000 | 0.0001 |
| **Mild with SSC** | 0.7575 | 0.1295 | 0.0063 | 0.1024 | 0.0023 | 0.0010 | 0.0008 | 0.0000 | 0.0001 |
| **Moderate** | 0.3539 | 0.2253 | 0.0157 | 0.3813 | 0.0128 | 0.0058 | 0.0047 | 0.0001 | 0.0004 |
| **Moderate with SSC** | 0.6739 | 0.1646 | 0.0086 | 0.1465 | 0.0035 | 0.0015 | 0.0013 | 0.0000 | 0.0001 |
| **Marked** | 0.0741 | 0.0934 | 0.0092 | 0.6800 | 0.0722 | 0.0359 | 0.0313 | 0.0009 | 0.0030 |
| **Marked with SSC** | 0.1627 | 0.1654 | 0.0144 | 0.5930 | 0.0339 | 0.0158 | 0.0132 | 0.0004 | 0.0012 |
| **Severe** | 0.0967 | 0.1153 | 0.0110 | 0.6658 | 0.0570 | 0.0276 | 0.0236 | 0.0007 | 0.0023 |
| **Severe with SSC** | 0.0164 | 0.0238 | 0.0026 | 0.5120 | 0.1766 | 0.1196 | 0.1305 | 0.0041 | 0.0143 |

$T=1$, $D=\text{Moderate}$, $S=\text{Splenectomized}$

| **From↓ To🡪** | **Mild** | **Mild with BP** | **Mild with SSC** | **Moderate** | **Moderate with SSC** | **Marked** | **Marked with SSC** | **Severe** | **Severe with SSC** |
| --- | --- | --- | --- | --- | --- | --- | --- | --- | --- |
| **Mild** | 0.7090 | 0.1506 | 0.0076 | 0.1273 | 0.0030 | 0.0013 | 0.0011 | 0.0000 | 0.0001 |
| **Mild with bone/joint pain** | 0.3978 | 0.2263 | 0.0150 | 0.3410 | 0.0107 | 0.0048 | 0.0039 | 0.0001 | 0.0004 |
| **Mild with SSC** | 0.5125 | 0.2129 | 0.0127 | 0.2493 | 0.0068 | 0.0030 | 0.0025 | 0.0001 | 0.0002 |
| **Moderate** | 0.1557 | 0.1610 | 0.0141 | 0.6015 | 0.0355 | 0.0166 | 0.0139 | 0.0004 | 0.0013 |
| **Moderate with SSC** | 0.4102 | 0.2259 | 0.0148 | 0.3302 | 0.0102 | 0.0046 | 0.0037 | 0.0001 | 0.0003 |
| **Marked** | 0.0262 | 0.0372 | 0.0039 | 0.6007 | 0.1467 | 0.0875 | 0.0863 | 0.0026 | 0.0089 |
| **Marked with SSC** | 0.0614 | 0.0798 | 0.0080 | 0.6809 | 0.0842 | 0.0429 | 0.0380 | 0.0011 | 0.0037 |
| **Severe** | 0.0348 | 0.0483 | 0.0051 | 0.6410 | 0.1256 | 0.0704 | 0.0663 | 0.0019 | 0.0067 |
| **Severe with SSC** | 0.0056 | 0.0083 | 0.0009 | 0.2807 | 0.1827 | 0.1797 | 0.2892 | 0.0115 | 0.0413 |

## Table A3. Comparison of Predicted with Observed Data

Percent of patients starting in mild health state, nonsplenectomized

|  | DS3 Score Study Data (Starting N=25) | | | | | |  | | Projected Data | | | | |  |
| --- | --- | --- | --- | --- | --- | --- | --- | --- | --- | --- | --- | --- | --- | --- |
| Year | N | Mild | Mild + BP | Mild + SSC | Mod | Mod + SSC |  | Mild | | Mild + BP | Mild + SSC | Mod | Mod + SSC | |
| 1 | 19 | 95 | 5 | 0 | 0 | 0 |  | 93 | | 4 | 0 | 3 | 0 | |
| 2 | 16 | 81 | 13 | 6 | 0 | 0 |  | 81 | | 10 | 0 | 8 | 0 | |
| 3 | 17 | 88 | 0 | 0 | 12 | 0 |  | 74 | | 12 | 1 | 12 | 0 | |
| 4 | 16 | 81 | 13 | 0 | 6 | 0 |  | 71 | | 12 | 1 | 14 | 0 | |
| 5 | 14 | 71 | 21 | 0 | 7 | 0 |  | 69 | | 13 | 1 | 15 | 1 | |
| 6 | 16 | 75 | 13 | 0 | 13 | 0 |  | 68 | | 13 | 1 | 16 | 1 | |
| 7 | 10 | 80 | 10 | 0 | 10 | 0 |  | 67 | | 13 | 1 | 16 | 1 | |
| 8 | 11 | 82 | 0 | 0 | 18 | 0 |  | 66 | | 13 | 1 | 16 | 1 | |
| 9 | 12 | 83 | 8 | 0 | 8 | 0 |  | 66 | | 13 | 1 | 16 | 1 | |
| 10 | 12 | 83 | 8 | 0 | 0 | 8 |  | 65 | | 13 | 1 | 16 | 1 | |

Frequencies for the marked, marked with SSC, severe, and severe with SSC health states = 0% for both predicted and observed data.

Percent of patients starting in moderate health state, nonsplenectomized

|  | DS3 Score Study Data (Starting N=31) | | | | | |  | Projected Data | | | | |
| --- | --- | --- | --- | --- | --- | --- | --- | --- | --- | --- | --- | --- |
| Year | N | Mild | Mild + BP | Mild + SSC | Mod | Mod + SSC |  | Mild | Mild + BP | Mild + SSC | Mod | Mod + SSC |
| 1 | 23 | 65 | 4 | 0 | 26 | 4 |  | 18 | 11 | 1 | 69 | 1 |
| 2 | 21 | 67 | 14 | 0 | 19 | 0 |  | 46 | 19 | 1 | 31 | 1 |
| 3 | 20 | 65 | 5 | 0 | 30 | 0 |  | 60 | 15 | 1 | 22 | 1 |
| 4 | 18 | 61 | 11 | 0 | 17 | 11 |  | 67 | 13 | 1 | 17 | 1 |
| 5 | 20 | 55 | 15 | 5 | 15 | 10 |  | 69 | 12 | 1 | 15 | 0 |
| 6 | 17 | 82 | 12 | 0 | 6 | 0 |  | 70 | 12 | 1 | 14 | 0 |
| 7 | 17 | 65 | 24 | 0 | 6 | 6 |  | 70 | 12 | 1 | 13 | 0 |
| 8 | 13 | 85 | 15 | 0 | 0 | 0 |  | 70 | 12 | 1 | 13 | 0 |
| 9 | 10 | 40 | 30 | 10 | 10 | 10 |  | 70 | 12 | 1 | 13 | 0 |
| 10 | 8 | 38 | 13 | 0 | 38 | 13 |  | 70 | 11 | 1 | 13 | 0 |

Frequencies for the marked, marked with SSC, severe, and severe with SSC health states = 0% for both predicted and observed data.

Starting in marked with SSC, nonsplenectomized

|  | DS3 Score Study Data (Starting N=17) | | | | | | | |  | Projected Data | | | | | | |
| --- | --- | --- | --- | --- | --- | --- | --- | --- | --- | --- | --- | --- | --- | --- | --- | --- |
| Year | N | Mild | Mild + BP | Mild + SSC | Mod | Mod + SSC | Marked | Marked + SSC |  | Mild | Mild + BP | Mild + SSC | Mod | Mod + SSC | Marked | Marked + SSC |
| 1 | 13 | 8 | 0 | 0 | 62 | 23 | 0 | 8 |  | 3 | 4 | 0 | 34 | 4 | 2 | 52 |
| 2 | 12 | 17 | 0 | 0 | 83 | 0 | 0 | 0 |  | 13 | 12 | 1 | 62 | 6 | 3 | 3 |
| 3 | 12 | 25 | 0 | 0 | 58 | 0 | 0 | 17 |  | 23 | 15 | 1 | 52 | 4 | 2 | 2 |
| 4 | 12 | 33 | 8 | 0 | 50 | 0 | 0 | 8 |  | 29 | 16 | 1 | 47 | 3 | 2 | 1 |
| 5 | 10 | 20 | 10 | 0 | 60 | 10 | 0 | 0 |  | 32 | 16 | 1 | 44 | 3 | 1 | 1 |
| 6 | 13 | 46 | 8 | 0 | 46 | 0 | 0 | 0 |  | 33 | 16 | 1 | 42 | 3 | 1 | 1 |
| 7 | 12 | 42 | 8 | 0 | 50 | 0 | 0 | 0 |  | 34 | 16 | 1 | 41 | 3 | 1 | 1 |
| 8 | 15 | 40 | 13 | 0 | 40 | 7 | 0 | 0 |  | 34 | 16 | 1 | 40 | 3 | 1 | 1 |
| 9 | 13 | 46 | 8 | 0 | 38 | 8 | 0 | 0 |  | 34 | 16 | 1 | 40 | 2 | 1 | 1 |
| 10 | 12 | 25 | 25 | 0 | 50 | 0 | 0 | 0 |  | 34 | 16 | 1 | 40 | 2 | 1 | 1 |

Frequencies for the severe and severe with SSC health states = 0% for both predicted and observed data.
